# Lipoprotein(a) in cardiovascular disease: pathophysiology, residual risk, and emerging therapeutic strategies

**DOI:** 10.3389/fmed.2026.1886347

**Published:** 2026-08-11

**Authors:** Lucio Giuseppe Granata, Simona Giubilato, Francesca Campanella, Francesco Amico, Stefania Angela Di Fusco, Furio Colivicchi

**Affiliations:** 1 UOC di UTIC con Cardiologia ed Emodinamica, AOE Cannizzaro, Catania, Italy; 2 Clinical and Rehabilitation Cardiology Unit, San Filippo Neri Hospital, ASL Roma 1, Rome, Italy

**Keywords:** antisense oligonucleotides, calcific aortic valve disease, cardiovascular prevention, lipoprotein(a), residual cardiovascular risk, RNA-based therapeutics, small interfering RNA

## Abstract

Lipoprotein(a) [Lp(a)] has emerged as a major, genetically determined, contributor to residual cardiovascular risk. Accumulating evidence from epidemiological studies, human genetics, and Mendelian randomization has unequivocally established elevated Lp(a) as an independent risk factor for atherosclerotic cardiovascular disease (ASCVD) and calcific aortic valve stenosis (CAVS). Lp(a) consists of a low-density lipoprotein-like particle in which apolipoprotein(a) is covalently linked to apolipoprotein B100. This unique structure confers proatherogenic, pro-inflammatory, and antifibrinolytic properties, largely mediated by oxidized phospholipids and the structural homology of apolipoprotein(a) with plasminogen. Circulating Lp(a) concentrations are genetically determined and remain relatively stable throughout life with minimal influence from lifestyle interventions. Conventional lipid-lowering therapies have little or no meaningful effect on circulating Lp(a) concentrations, leaving an important component of residual cardiovascular risk unaddressed. In contrast, RNA-based therapeutics targeting hepatic *LPA* expression have demonstrated reductions in circulating Lp(a) of up to 80–90% and are currently being evaluated in phase 3 cardiovascular outcome trials. This narrative review summarizes the molecular biology, genetics, epidemiology, pathophysiological mechanisms, and clinical relevance of Lp(a), and critically examines current and emerging therapeutic strategies aimed at reducing Lp(a)-mediated cardiovascular risk.

## Graphical abstract

**Figure:**
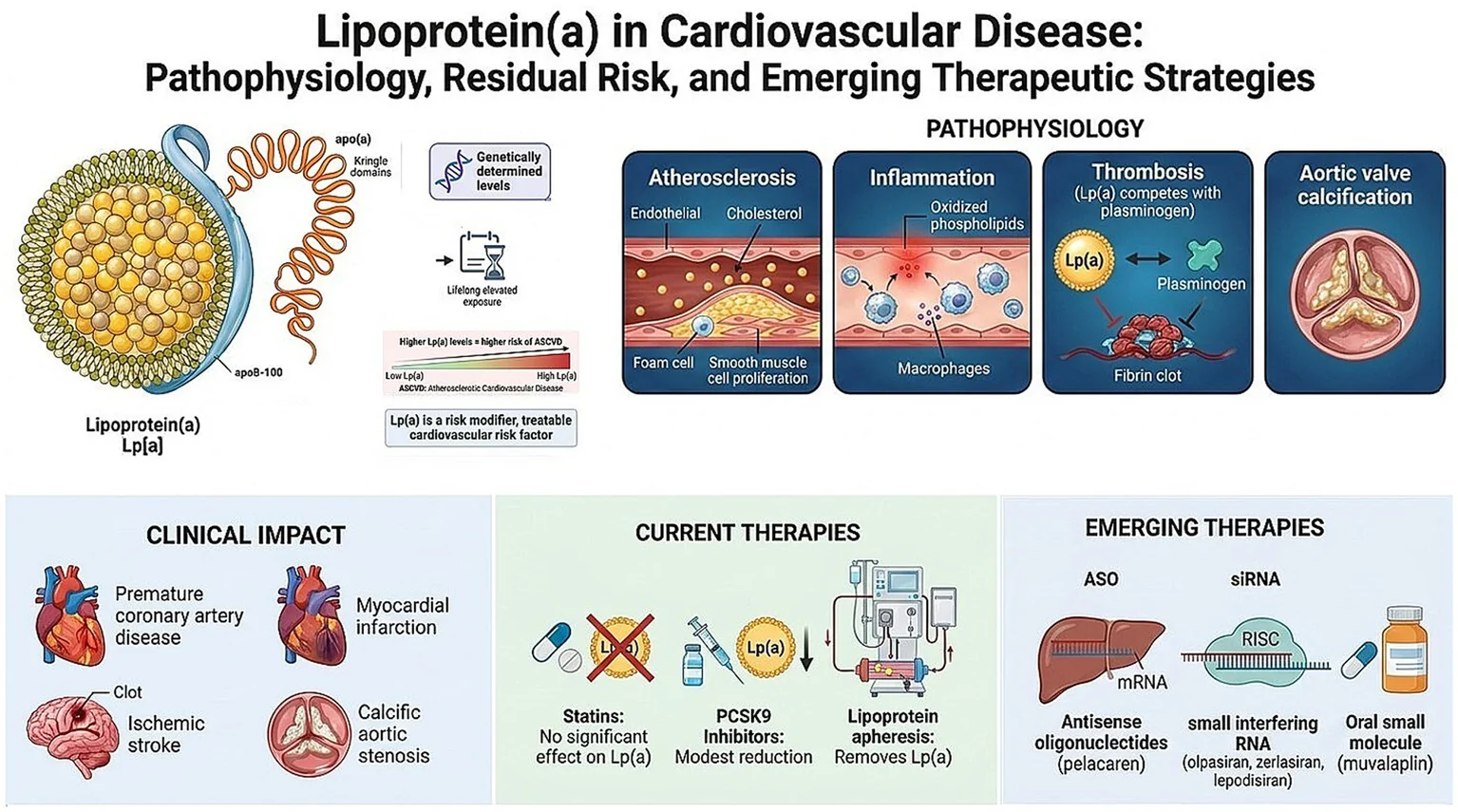

## 1 Introduction

Although first identified in the early 1960s, Lp(a) remained for many years a largely overlooked lipoprotein fraction, mainly due to the absence of effective therapies and the complexity of its genetic regulation. This paradigm has changed substantially following the convergence of epidemiological evidence, genetic association studies, and Mendelian randomization analyses, which have demonstrated that elevated Lp(a) levels modify the atherosclerotic cardiovascular disease (ASCVD) risk and are associated with calcific aortic valve disease (1–3). Functionally, it combines the atherogenic properties of LDL apo(B) containing lipoproteins with additional pro-inflammatory, pro-oxidative, and antifibrinolytic effects largely mediated by apo(a) and its carriage of oxidized phospholipids (4, 5). Circulating Lp(a) concentrations are largely genetically determined and show minimal responsiveness to lifestyle modification or conventional lipid-lowering therapies (1, 3, 6, 7). Large-scale human genetic studies have strengthened the evidence supporting a causal role of Lp(a) in ASCVD (8). Consequently, elevated Lp(a) represents a major contributor to cardiovascular risk that may persist even in patients achieving guideline-recommended LDL-C targets (9, 10). Moreover, accounting for the cholesterol content carried by Lp(a) may refine lipid risk assessment by enabling more accurate estimation of “corrected” low-density lipoprotein cholesterol (LDL-C) in individuals with elevated Lp(a) (1, 11). The clinical relevance of Lp(a) extends beyond coronary artery disease (CAD) to include ischemic stroke, peripheral artery disease, and, most notably, calcific aortic valve stenosis (12–17). The development of emerging RNA-based Lp(a)-targeted therapies has provided proof-of-concept that pharmacological Lp(a) lowering can selectively reduce circulating Lp(a) concentrations by up to 90%, thereby reshaping the therapeutic landscape and transforming Lp(a) from a largely “non-modifiable” cardiovascular risk marker into a promising therapeutic target (1, 18).

## 2 Molecular structure and genetic regulation of lipoprotein(a)

Lp(a) was first described by Berg in 1963 as an LDL-related serum factor and is now recognized as a distinct lipoprotein particle in which the apo(a) component confers unique biological properties (7, 19). It consists of an LDL-like core particle containing cholesteryl esters and triglycerides, with apoB100 forming the structural backbone to which apo(a) is covalently attached through a disulfide bond. Apo(a) is a large, highly polymorphic glycoprotein characterized by multiple kringle domains, particularly kringle IV (KIV) repeats. Among these, the kringle IV type 2 (KIV-2) region represents a highly polymorphic domain consisting of tandemly repeated units due to a variable number of copies encoded by the *LPA* gene; the number of repeats varies markedly between individuals, generating apo(a) isoforms of different sizes. Apo(a) is synthesized independently by hepatocytes following *LPA* gene expression and is subsequently assembled extracellularly, or at the hepatocyte surface, with a pre-existing apoB100-containing LDL particle through a single covalent disulfide bond. This extracellular assembly step is fundamental to the biology of Lp(a) and provides the mechanistic rationale for both RNA-targeting therapies, which suppress apo(a) synthesis, and assembly-targeting agents (20, 21). The number of KIV-2 repeats represents the primary determinant of plasma Lp(a) concentrations (7, 22, 23). Smaller apo(a) isoforms, characterized by fewer KIV-2 repeats, are typically associated with higher circulating Lp(a) levels, whereas larger isoforms correlate with lower concentrations, likely reflecting differences in hepatic synthesis and secretion efficiency. Consequently, an inverse relationship exists between apo(a) isoform size and circulating Lp(a) levels (7, 22, 24, 25). This structural variability partly explains the marked interindividual variability in Lp(a) concentrations observed in the general population (25, 26). Importantly, more than 90% of this variability is attributable to genetic variation at the *LPA* locus on chromosome 6, making Lp(a) one of the most strongly genetically determined cardiovascular risk factors identified to date (8, 27). Each individual inherits two *LPA* alleles, which frequently differ in their KIV-2 copy number, ranging from approximately 1 to more than 40 copies per allele, therefore the combined contribution of both alleles is a major determinant of apo(a) isoform size and circulating Lp(a) concentrations (28, 29). The key structural features and functional properties of lipoprotein(a) are summarized in Table 1.

**Table 1:** Structural and biological characteristics of lipoprotein(a).

Feature	Description	Clinical relevance
Core structure	LDL-like particle containing cholesterol esters	Atherogenic lipid transport
Apolipoprotein(a)	Covalently bound to apoB100	Unique biological properties
Genetic regulation	Determined by *LPA* locus (kringle IV repeats)	>90% heritability
Plasma stability	Stable across lifespan	Single lifetime measurement sufficient
Lifestyle influence	Minimal	Not modifiable by diet/exercise
Oxidized phospholipids	Preferentially carried by Lp(a)	Pro-inflammatory, pro-calcific
Fibrinolysis interference	Apo(a) competes with plasminogen	Pro-thrombotic risk
Main disease associations	CAD, MI, stroke, aortic stenosis	Independent causal risk

Summary of the main structural features, genetic determinants, biological properties, and clinical relevance of Lp(a). Apo(a), apolipoprotein(a); apoB100, apolipoprotein B100; CAD, coronary artery disease; LDL, low-density lipoprotein; Lp(a), lipoprotein(a); MI, myocardial infarction.

## 3 Pathophysiological mechanisms linking Lp(a) to cardiovascular disease

**Figure 1:**
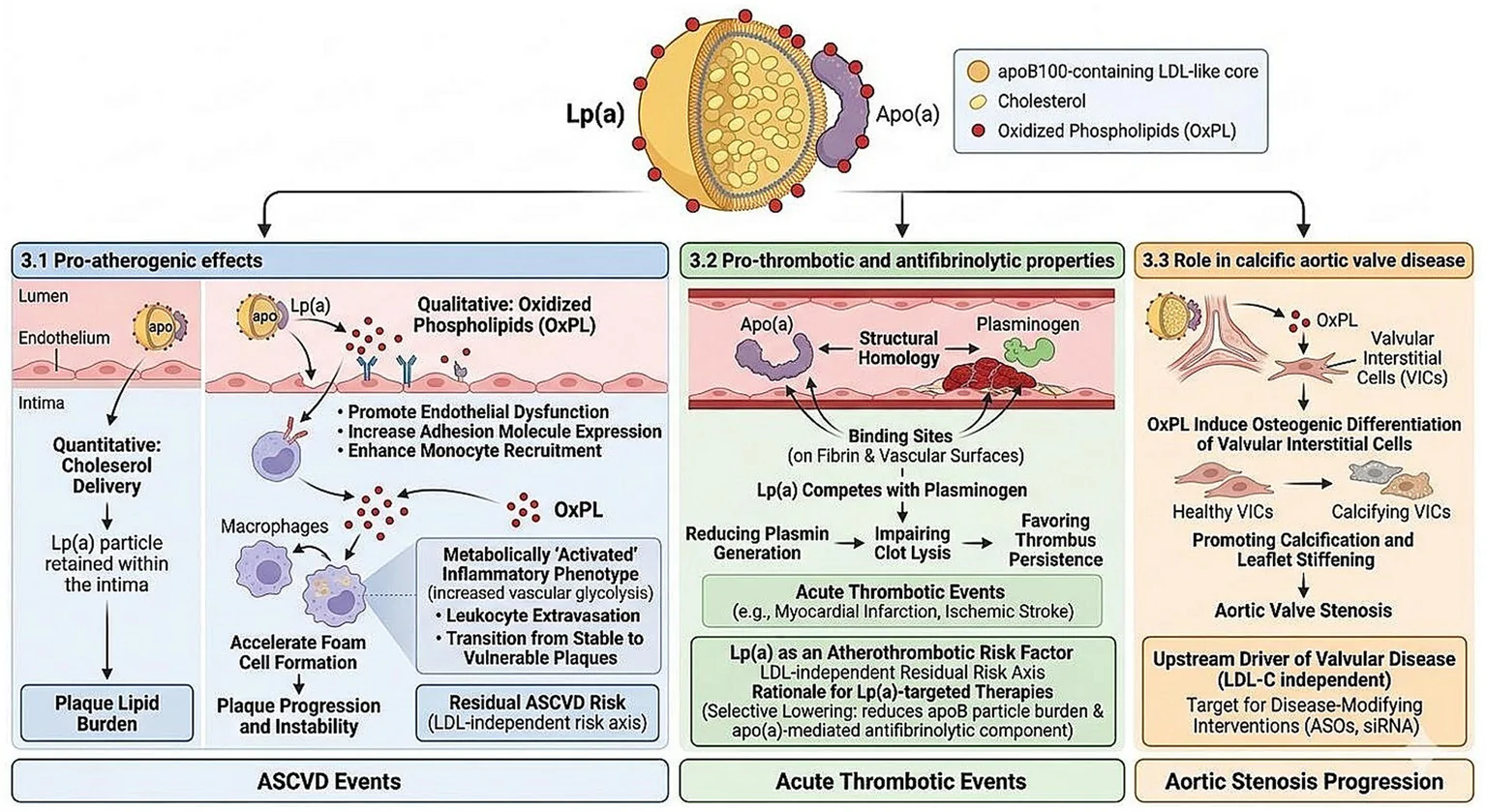
Pathophysiological effects of lipoprotein(a): proatherogenic, prothrombotic, and pro-calcific mechanisms. The figure summarizes the major pathogenic pathways linking lipoprotein(a) [Lp(a)] to cardiovascular disease. Lp(a) promotes atherogenesis through arterial wall retention, endothelial dysfunction, oxidative phospholipid cargo, vascular inflammation, and foam-cell formation. Its structural homology with plasminogen and the presence of oxidized phospholipids contribute to impaired fibrinolysis, enhanced thrombogenicity, and amplification of thrombo-inflammatory pathways. In addition, Lp(a) has been implicated in the initiation and progression of calcific aortic valve disease by promoting valvular interstitial cell osteogenic differentiation, inflammatory signaling, and calcium deposition within the aortic valve leaflets.

Beyond being a genetically determined cardiovascular risk factor, Lp(a) exerts pleiotropic pathogenic effects through proatherogenic, prothrombotic, and pro-calcific mechanisms that drive both ASCVD and calcific aortic valve stenosis (CAVS) (Figure 1).

### 3.1 Pro-atherogenic effects

Lp(a) promotes atherogenesis through both quantitative and qualitative mechanisms. Similar to LDL, its apoB100-containing core transports cholesterol into the arterial wall, where it crosses the endothelium by transcytosis and is retained within the intima, contributing directly to lipid accumulation in atherosclerotic plaques (5). In addition to this LDL-like effect, Lp(a) possesses unique proatherogenic properties largely mediated by oxidized phospholipids (OxPLs). Lp(a) is the major plasma carrier of OxPLs, which are preferentially associated with the apo(a) component rather than the LDL-like apoB100-containing particle (3, 27). These apo(a)-associated OxPLs induce endothelial dysfunction, upregulate vascular adhesion molecules, promote monocyte recruitment, and enhance macrophage foam-cell formation, thereby amplifying vascular inflammation and plaque progression. Experimental studies further suggest that apo(a)-associated OxPLs promote a metabolically activated inflammatory phenotype characterized by increased vascular glycolysis and enhanced leukocyte extravasation, favoring the transition from stable to vulnerable plaques (30, 31). Consistent with these experimental observations, coronary computed tomography angiography (CCTA) studies have shown that elevated Lp(a) concentrations are associated with greater plaque burden, high-risk plaque characteristics, and an increased incidence of cardiovascular events, with much of this excess risk appearing to be mediated by inflammatory and vulnerable plaque phenotypes (32–34). A recent PIONEER IV substudy including 392 coronary vessels from 75 propensity-matched pairs demonstrated that elevated Lp(a) levels were associated with greater epicardial flow limitation and a more diffuse coronary atherosclerotic phenotype. Specifically, patients had a higher prevalence of functionally significant coronary flow limitation (QFR ≤ 0.80: 31% vs. 19%) and lower QFR-derived pressure pullback gradient index (QFR-PPGi) values, consistent with a diffuse rather than focal pattern of coronary atherosclerosis, thus extending the pathogenic role of Lp(a) beyond plaque burden, and suggesting that elevated levels may promote diffuse CAD and impaired epicardial coronary conductance (35). Intracoronary imaging data further support the association between elevated Lp(a) and plaque vulnerability. In a *post hoc* analysis of the YELLOW II study, patients in the highest Lp(a) quartile exhibited significantly thinner fibrous caps on optical coherence tomography (OCT), a well-established marker of vulnerable plaque phenotype. Although high-intensity rosuvastatin therapy increased fibrous cap thickness overall despite a modest increase in circulating Lp(a), changes in Lp(a) concentrations were not associated with fibrous cap modification, suggesting that baseline Lp(a) may reflect plaque vulnerability rather than short-term plaque stabilization (36).

Finally, in selected populations, elevated Lp(a) predicts cardiovascular events independently of high-sensitivity C-reactive protein (hs-CRP), while concomitant elevation of both biomarkers identifies individuals with particularly high residual atheroinflammatory risk (37–40).

Collectively, these findings indicate that Lp(a) promotes atherosclerosis not only by increasing cholesterol deposition but also by amplifying vascular inflammation and plaque vulnerability, thereby contributing to residual cardiovascular risk despite intensive LDL-C lowering (5).

### 3.2 Pro-thrombotic and antifibrinolytic properties

Beyond the proatherogenic effects, apo(a)-associated OxPLs are also thought to contribute to the antifibrinolytic and pro-thrombotic properties of Lp(a). The pro-thrombotic effect arise primarily from the structural organization of apo(a). Apo(a) shares extensive homology with plasminogen through its kringle domains but lacks an active protease domain. This structural mimicry enables apo(a) to compete with plasminogen for binding sites on fibrin, endothelial cells, platelets, and extracellular matrix proteins without generating plasmin; consequently, plasmin formation is reduced, fibrin degradation is impaired, and thrombus persistence is favored (8, 27, 41). This antifibrinolytic mechanism is supported by intracoronary imaging studies. Optical coherence tomography (OCT) performed during acute coronary syndrome has demonstrated that elevated hyperacute-phase Lp(a) concentrations are associated with larger intracoronary thrombus burden, whereas lower stable-phase concentrations are associated with layered plaque, suggesting a role for Lp(a) in both thrombus formation and plaque healing (42). Although these mechanisms are biologically well established, evidence for a direct causal thrombotic effect derives mainly from experimental studies. Human genetic and clinical studies suggest that the cardiovascular risk associated with Lp(a) is mediated predominantly through the interplay between atherogenesis, vascular inflammation, and impaired fibrinolysis rather than thrombosis alone (5, 43, 44). These complementary mechanisms explain the consistent epidemiological association between elevated Lp(a) concentrations and atherothrombotic events, supporting the concept of Lp(a) as a mediator of atherothrombosis rather than simply a lipid biomarker (2, 7, 27). They also provide the biological rationale for selective Lp(a)-lowering therapies, which may simultaneously reduce cholesterol delivery to the arterial wall and apo(a)-mediated inhibition of fibrinolysis, thereby targeting an important component of residual cardiovascular risk (3).

### 3.3 Role in calcific aortic valve disease

Among the most clinically relevant and emerging aspects of Lp(a) biology is its role in CAVS (6, 27, 45). Multiple observational and genetic studies have demonstrated a strong association between elevated Lp(a) levels and both the presence and progression of aortic valve calcification (16, 46–48). Mechanistically, OxPLs transported by Lp(a) are thought to promote osteogenic differentiation of valvular interstitial cells, thereby driving progressive valvular calcification and leaflet stiffening (17, 49, 50). These processes involve the activation of pro-calcific signaling pathways within the aortic valve, ultimately leading to extracellular matrix remodeling and mineral deposition. Notably, the association between Lp(a) and CAVS appears largely independent of traditional cardiovascular risk factors and LDL-C levels, supporting the concept that Lp(a) functions as an upstream driver of valvular disease rather than merely reflecting global cardiometabolic risk (6, 46). Large population-based data from the United Kingdom Biobank further support this role, showing that elevated Lp(a) was independently associated with incident CAVS, with additional sex-specific effects beyond traditional lipid markers; moreover, it improved risk prediction when added to ApoB or LDL-C, reinforcing the role as an independent risk enhancer in aortic valve stenosis pathobiology and risk stratification (51). These observations have important clinical implications, as they identify this biomarker as a potential disease-modifying target in a condition currently treated predominantly with surgical or transcatheter valve replacement. The Lp(a)-CAVS axis therefore represents one of the strongest rationales for the development of pharmacological therapies aimed at slowing disease progression. In this context, the rapidly expanding pipeline of Lp(a)-lowering agents, including antisense oligonucleotides and small interfering RNAs (siRNA), may provide novel opportunities to modify the natural history of CAVS (17, 45). A long-term observational study in patients undergoing chronic lipoprotein apheresis showed that individuals with elevated Lp(a) exhibited more severe aortic valve disease at baseline, supporting the association between this biomarker and calcific aortic stenosis. However, during prolonged follow-up, progression of aortic stenosis was comparable regardless of Lp(a) levels, suggesting that lipoprotein apheresis may help attenuate valve disease progression in high-risk patients (52). Therefore, Lp(a) has emerged as a central mediator linking lipid-driven inflammation to valvular calcification and disease progression.

### 3.4 Emerging associations

Beyond myocardial infarction, stroke and CAVS, elevated Lp(a) has been linked to a broader spectrum of cardiovascular and systemic conditions, including peripheral artery disease, heart failure and chronic kidney disease supported by increasingly data (53–57). By contrast, associations with atrial fibrillation, mitral annular calcification, abdominal aortic aneurysm and venous thromboembolism remain less consistent (48, 58–62). Moreover, coronary microvascular dysfunction (CMD) is increasingly recognized as a major contributor to myocardial ischemia, particularly in patients with angina or ischaemia and non-obstructive coronary arteries (ANOCA/INOCA) (63). Beyond its established role in epicardial atherosclerosis, emerging evidence suggests that Lp(a) may contribute to CMD through endothelial dysfunction, oxidative stress, inflammation, microvascular remodeling, and microthrombotic pathways driven by oxidized phospholipids and the antifibrinolytic properties of apolipoprotein(a) (64). Consistently, elevated Lp(a) levels (≥50 mg/dL) has been associated with a trend toward higher prevalence of CMD and lower coronary flow reserve, supporting a potential role in coronary microvascular impairment, although larger studies are still needed to confirm this association (65). The major cardiovascular and valvular conditions associated with elevated Lp(a) are summarized in Table 2.

**Table 2:** Cardiovascular and valvular conditions associated with elevated lipoprotein(a).

Condition	Strength of association	Pathophysiological link
Premature coronary artery disease	Strong	Accelerated atherogenesis
Myocardial infarction	Strong	Atherothrombosis
Ischemic stroke	Moderate–strong	Thrombosis and plaque instability
Recurrent ASCVD despite low LDL-C	Strong	Residual genetic risk
Calcific aortic valve stenosis	Very strong	OxPL-driven osteogenic signaling
Polyvascular disease	Moderate	Systemic inflammatory burden
Familial hypercholesterolemia	Frequent coexistence	Risk amplification

Summary of the principal cardiovascular and valvular disorders consistently associated with elevated Lp(a) and their predominant underlying pathophysiological mechanisms. ASCVD, atherosclerotic cardiovascular disease; LDL-C, low-density lipoprotein cholesterol; Lp(a), lipoprotein(a); OxPL, oxidized phospholipids.

The key molecular and pathophysiological mechanisms underlying Lp(a)-mediated cardiovascular risk are summarized in Table 3.

**Table 3:** Molecular and pathophysiological mechanisms linking lipoprotein(a) to cardiovascular disease.

Domain	Molecular mechanism	Downstream effect	Clinical implication
Cholesterol transport	LDL-like lipid core	Cholesterol accumulation in arterial intima	Accelerated atherosclerosis
Apo(a)–plasminogen homology	Competitive inhibition of plasminogen binding	Reduced fibrinolysis	Pro-thrombotic milieu
Oxidized phospholipids (OxPL)	Lp(a) as primary plasma carrier	Endothelial activation, monocyte recruitment	Plaque inflammation
Inflammatory signaling	Activation of TLRs and NF-κB pathways	Cytokine release, vascular inflammation	Plaque vulnerability
Smooth muscle cell effects	OxPL-induced phenotypic switching	Intimal hyperplasia	Disease progression
Valvular interstitial cell activation	Osteogenic differentiation via OxPL	Valve calcification	Aortic stenosis development
Genetic pleiotropy	*LPA* variants affecting apo(a) size	High lifelong exposure	Early-onset disease

Molecular mechanism, downstream effect, and clinical implication for each pathogenic domain.

## 4 Epidemiology, population and sex distribution

Lp(a) concentrations exhibit a markedly right-skewed distribution in the general population (26). It is reasonable to consider elevated Lp(a) levels >50 mg/dL (≥105 nmol/L), affecting approximately 20% of individuals, in order to refine CV risk estimation across the spectrum of CV risk; moreover, this cut-off level should be considered as a risk modifier to potentially reclassify the CV risk category specifically in individuals at moderate risk or individuals close to treatment decision thresholds (6, 27). Pronounced ethnic differences in Lp(a) concentrations and prognostic thresholds have been consistently reported, highlighting Lp(a) as one of the most variable lipoprotein traits across populations (66, 67). Individuals of African ancestry tend to have substantially higher median Lp(a) levels than those of European or Asian descent. These differences are only partly explained by conventional risk factors and largely reflect the underlying genetic architecture at the *LPA* locus (66, 68). Despite these variations in absolute distribution, the relative association between Lp(a) and cardiovascular outcomes appears broadly consistent across ethnic groups, suggesting that elevated Lp(a) represents a clinically meaningful risk signal across ancestries rather than a population-specific phenomenon (27, 66).

These epidemiological observations have direct implications for clinical practice. First, they caution against a uniform interpretation of absolute cut-offs when assessing risk across diverse populations and underscore the importance of interpreting Lp(a) levels within ancestry-informed distributions (66, 69). In addition, sex differences in Lp(a)-related risk are increasingly recognized. Although Lp(a) is associated with ASCVD in both sexes (70), recent data suggest that its clinical impact may be modulated by sex, age, hormonal status and the interaction with other lipid traits (71). In particular, it appears to confer excess cardiovascular risk in both women and men, but some studies indicate different risk patterns across the lifespan, with post-menopausal women showing increasing vulnerability and men showing stronger associations with coronary plaque burden and adverse plaque features (72, 73). Among patients with familial hypercholesterolemia, women with CAD exhibited significantly higher median Lp(a) concentrations than those without CAD, supporting the concept that Lp(a) may be a particularly important determinant of cardiovascular risk in women (74). Moreover, high Lp(a) level may attenuate the apparently protective effect of high HDL-C, with relevant sex-specific differences, supporting the need to incorporate Lp(a) into sex-sensitive cardiovascular risk stratification (74, 75).

Notably, the diagnosis of elevated Lp(a) may represent a source of psychological distress. In the prospective ELITE study, among 5,726 partecipants, approximately 40% of individuals with elevated Lp(a) reported concern or anxiety after becoming aware of their Lp(a) status, highlighting its potential psychosocial impact (76). Given the increasing emphasis on Lp(a) screening in women, individualized counseling and education should be integrated into comprehensive cardiovascular risk management to improve adherence to preventive strategies while minimizing unnecessary anxiety and psychological distress (77).

## 5 Clinical evidence linking Lp(a) to cardiovascular outcomes

Large prospective cohort studies and meta-analyses have consistently shown that elevated Lp(a) levels are independently associated with a higher risk of CAD myocardial infarction, peripheral artery disease, and ischemic stroke, even after adjustment for LDL-C and other traditional risk factors (7, 15, 27, 76). Individual-participant meta-analyses from long-term prospective cohorts further support a continuous relationship between long-term Lp(a) concentrations and vascular outcomes, indicating that risk increases across the entire distribution rather than only at extreme levels (27, 78). Similarly, pooled analyses indicate that elevated Lp(a) is associated with an increased risk of ischemic stroke, reinforcing its relevance beyond coronary endpoints (12, 79).

Genetic studies further support a causal relationship between Lp(a) and cardiovascular disease, with genetic variants associated with higher circulating Lp(a) concentrations conferring proportional increases in cardiovascular risk (7, 24, 68). Notably, contemporary evidence suggests that, on an equimolar basis, Lp(a) may exert a greater atherogenic potential than LDL particles, reflecting the combined contribution of apoB-mediated arterial retention and apo(a)/OxPL-driven inflammatory and thrombotic pathways (80).

Clinically, elevated Lp(a) is frequently observed in patients with premature ASCVD, recurrent cardiovascular events, or persistent residual risk despite intensive LDL-C lowering, supporting the concept of Lp(a) as an independent, LDL-C–unrelated contributor to cardiovascular risk (7, 27). In familial hypercholesterolemia, concomitant elevation of Lp(a) may further amplify lifetime atherothrombotic exposure and help explain event rates that appear disproportionate to achieved LDL-C levels (81). In addition, *post hoc* analyses from randomized outcome trials of LDL-lowering therapies suggest that treatments producing modest reductions in Lp(a) may yield greater absolute benefit among individuals with higher baseline levels, supporting a potential “risk enrichment” strategy for future targeted trials (18, 80).

Recent observational evidence suggests that the cardiovascular risk associated with markedly elevated Lp(a) may not be constant over time. In a cohort of patients with previous cardiovascular disease, subjects with Lp(a) concentrations ≥90 mg/dL experienced the highest relative risk of recurrent major adverse cardiovascular events during the first 18–24 months after measurement, with progressively attenuated hazard ratios during longer follow-up. These findings support the concept that elevated Lp(a) may identify patients at particularly high short-term residual cardiovascular risk; whether these observations should influence the timing of Lp(a) assessment or justify repeated measurements in selected clinical settings remains to be determined (82).

The convergence of epidemiologic, genetic, and clinical trial evidence has reinforced interest in dedicated Lp(a)-lowering strategies, including antisense oligonucleotide and siRNA approaches, currently under investigation in phase 3 outcome trials to determine whether substantial Lp(a) reduction translates into improved cardiovascular outcomes (27, 80).

## 6 Measurement of lipoprotein(a): clinical indications and practical considerations

Because Lp(a) levels are largely genetically determined and remain relatively stable throughout life, major professional societies recommend measuring Lp(a) at least once in every adult to detect inherited elevation and refine cardiovascular risk classification (6, 69, 83, 84). Lp(a) measurement is particularly recommended in individuals with:

- Premature ASCVD (6, 85).
- Family history of early-onset CAD or sudden cardiac death (83, 86, 87).
- Recurrent cardiovascular events despite optimal LDL-C control (88, 89).
- CAVS, particularly when occurring at a younger age (16, 46).
- Diabetes mellitus type II (90).
- Women above 50 years old (71, 91).
- History of familiar hypercholesterolemia (74).
- Elevated LDL-C levels (92).
- Apparent discordance between traditional risk factors and observed clinical risk (7).

From a preventive perspective, measuring Lp(a) allows identification of individuals whose cardiovascular risk may be underestimated by conventional risk scores (1, 7, 69).

Recent population-based evidence has strengthened the rationale for routine Lp(a) assessment in primary prevention. In the EPIC-Norfolk study, involving more than 17,000 initially healthy European adults followed for 20 years, a single baseline measurement of LDL-C, hsCRP, and Lp(a) independently predicted future major adverse cardiovascular events (MACEs). Importantly, the combination of these three biomarkers provided additive prognostic information, supporting the concept of universal one-time screening to improve lifetime cardiovascular risk stratification (93).

Of note, in patients with type 2 diabetes mellitus, elevated Lp(a) identifies a subgroup at particularly high residual cardiovascular risk and has been shown to predict adverse coronary outcomes independently of other traditional risk factors (90, 94, 95). High Lp(a) levels in diabetic patients are associated with high coronary artery plaque burden at baseline and low attenuation volume progression (96).

A second measurement is generally unnecessary for routine risk prediction but may be appropriate in selected circumstances where Lp(a) levels may be transiently altered or where documenting change is clinically relevant. These include situations in which the initial measurement was obtained during acute inflammatory states or infection, in the presence of significant kidney or liver disease, during pregnancy, after menopause, or after major changes in renal or hepatic status (6, 83, 97, 98).

Observational data indicate that Lp(a) concentrations increase by approximately 20–30% as women transition from premenopause to postmenopause. These findings have prompted reconsideration of the “once-in-a-lifetime” testing approach in women and suggest that reassessment after menopause may be clinically informative, particularly when Lp(a) exceeds 50 mg/dL (≈105 nmol/L) (72).

Repeat testing may also be appropriate after initiating Lp(a)-lowering interventions, such as lipoprotein apheresis or emerging Lp(a)-targeted therapies, to confirm biological response and guide management.

Measurement of Lp(a) can also be informative in the diagnostic work-up of suspected familial hypercholesterolemia (FH). Cholesterol carried within Lp(a) [Lp(a)-C] contributes to measured LDL-C levels in most routine assays, potentially influencing clinical scoring systems such as the Dutch Lipid Clinic Network (DLCN) criteria and supporting a more nuanced interpretation of markedly elevated LDL-C phenotypes (99).

Moreover, in a multicenter retrospective study of 1,581 Japanese patients with established CAD (59.5% acute coronary syndromes and 85.7% with multivessel disease), Kataoka et al. (100) evaluated whether Lp(a)-associated residual cardiovascular risk persists despite achievement of current guideline-recommended LDL-C goals. Over a median follow-up of 5.1 years, higher Lp(a) levels were associated with a stepwise increase in MACE, defined as cardiac death, non-fatal myocardial infarction, or non-culprit revascularization. Importantly, this relationship was not abolished by intensive LDL-C control. Among patients with LDL-C < 55 mg/dL, event rates increased from 1.4 to 4.7 and 7.5 events per 100 person-years across Lp(a) categories of <30, 30–49, and ≥50 mg/dL, respectively. Accordingly, the estimated 5-year MACE risk rose from 5.0 to 17.0 and 33.4% across the same Lp(a) strata. These observations support the concept that Lp(a) represents an independent and clinically relevant driver of residual cardiovascular risk, even among patients achieving contemporary LDL-C targets. Furthermore, the results suggest that Lp(a) levels of approximately 30 mg/dL may already identify patients at increased cardiovascular risk, with a progressive increase in MACE observed at higher concentrations, particularly at levels ≥50 mg/dL.

Finally, knowledge of elevated Lp(a) levels may provide additional rationale for considering PCSK9 inhibitor therapy when treatment intensification is indicated. PCSK9 monoclonal antibodies produce potent LDL-C reductions and consistently lower Lp(a) concentrations by approximately 20–30%. *Post hoc* analyses suggest that individuals with higher baseline Lp(a) may derive greater absolute benefit from these therapies, supporting their integration into comprehensive prevention strategies while awaiting results from Lp(a)-specific outcome trials (88, 89).

### 6.1 Units of measurement and interpretation

Lp(a) concentrations can be reported either as mass concentration (mg/dL) or molar concentration (nmol/L). However, these units are not directly interchangeable because apo(a) isoform size substantially influences particle mass, whereas molar concentration more closely reflects particle number. Consequently, the commonly cited conversion factor between mg/dL and nmol/L is unreliable, as the relationship varies according to apo(a) isoform distribution and assay calibration (5, 69, 84). Applying a fixed conversion factor may therefore result in clinically relevant misclassification. For this reason, Lp(a) measurement and reporting in nmol/L using isoform-insensitive assays is generally preferred when available (20, 38, 44). Mass-based assays may overestimate or underestimate Lp(a) concentrations depending on apo(a) isoform size, whereas molar reporting better reflects particle number (62, 69). Despite these limitations, several pragmatic thresholds are widely used in clinical practice, the risk of major CV events starts to slightly increase in individuals with Lp(a) levels >62 nmol/L (30 mg/dL), and the increase in risk becomes more pronounced in individuals with Lp(a) levels ≥105 nmol/L (50 mg/dL) (83, 101, 102). Importantly, cardiovascular risk associated with Lp(a) increases continuously across its distribution rather than abruptly above a single threshold (78, 103).

The principal analytical limitations and clinical implications of Lp(a) measurement are summarized in Table 4.

**Table 4:** Analytical and clinical considerations for Lp(a) measurement.

Aspect	Technical detail	Clinical relevance
Units of reporting	mg/dL vs. nmol/L	Not directly interchangeable
Apo(a) isoform dependency	Assay sensitivity influenced by kringle IV repeats	Risk of misclassification
Standardization	ISO/IFCC reference materials	Inter-laboratory variability persists
Biological variability	Minimal intra-individual variation	Single lifetime measurement sufficient
Acute phase response	Mild increase during inflammation	Avoid measurement during acute illness
Threshold interpretation	Continuous risk, no absolute cut-off	Risk stratification rather than diagnosis
Ethnic variability	Higher levels in African ancestry	Population-specific risk assessment
Clinical timing	Preferably in stable outpatient setting	Improves interpretability

Summary of the principal analytical limitations and practical considerations for interpretation of Lp(a) measurements. ISO, International Organization for Standardization; IFCC, International Federation of Clinical Chemistry and Laboratory Medicine.

## 7 Impact of currently available lipid-lowering therapies on Lp(a)

### 7.1 Introductive overview

Most currently available lipid-lowering therapies (LLT) have little or no clinically meaningful effect on Lp(a) concentrations (5, 80). This limitation helps explain why Lp(a)-mediated cardiovascular risk may persist even under intensive LDL-C reduction (86, 101). Overall, currently available LLT provide only a partial solution for Lp(a)-mediated risk, reinforcing the need for dedicated Lp(a)-lowering strategies (6, 104, 105).

### 7.2 Statins

Statins remain the cornerstone of ASCVD prevention because they effectively reduce LDL-C levels and improve cardiovascular outcomes in both primary and secondary prevention settings (83). However, their effect on Lp(a) concentrations is limited. Overall, statins appear largely neutral with respect to Lp(a) and may even produce a modest increase in circulating levels in some patients (5). In the most comprehensive systematic review and meta-analysis available, statin therapy was associated with a small upward shift in Lp(a), generally within the single-digit to low–double-digit percentage range depending on baseline Lp(a) levels and study characteristics (106). The mechanisms underlying this effect remain incompletely understood. Importantly, this observation does not reduce the well-established cardiovascular benefit of statins, as the magnitude of risk reduction mediated by LDL-C lowering clearly outweighs any small increase in Lp(a). Rather, it underscores the fact that statins do not directly address Lp(a)-related risk. Consequently, patients with elevated baseline Lp(a) may require broader strategies to manage residual cardiovascular risk beyond LDL-C lowering alone (80, 106).

### 7.3 Ezetimibe

Ezetimibe lowers LDL-C primarily by inhibiting intestinal cholesterol absorption through blockade of the Niemann–Pick C1-like 1 (NPC1L1) transporter. Its impact on Lp(a) concentrations, however, appears minimal (5). Meta-analyses of randomized trials indicate that average reduction in Lp(a) associated with ezetimibe is generally small, typically <5–10%, which are unlikely to produce clinically meaningful changes in Lp(a)-mediated cardiovascular risk (107, 108). Consequently, the clinical role of ezetimibe in patients with elevated Lp(a) is largely indirect. In practice, ezetimibe is primarily used to intensify LDL-C lowering often in combination with statins to further reduce apoB-containing particle burden and overall cardiovascular risk. However, Lp(a)-related residual risk is expected to remain largely unaffected by ezetimibe therapy (104, 105).

### 7.4 PCSK9 inhibitors

Monoclonal antibodies targeting PCSK9, including evolocumab and alirocumab, represent the only currently available pharmacological therapies that consistently produce a moderate reduction in Lp(a) concentrations, typically in the range of approximately 20–30%, in addition to their potent LDL-C-lowering effects (109, 110).

Phase II pooled analyses and subsequent larger clinical programs have consistently demonstrated reductions in Lp(a) alongside marked decreases in LDL-C and apoB levels (59). Importantly, *post hoc* analyses from large outcome trials suggest that higher baseline Lp(a) levels may identify patients who derive greater absolute cardiovascular benefit from PCSK9 inhibition, supporting the concept that Lp(a) contributes to residual risk even when LDL-C is aggressively lowered (89).

Further analyses from the ODYSSEY OUTCOMES trial suggested that reductions in Lp(a) were associated with lower cardiovascular event rates in a manner not fully explained by LDL-C lowering alone, indicating that at least part of the benefit may be mediated through Lp(a) reduction (111). Nevertheless, PCSK9 inhibitors are not currently approved specifically for the treatment of elevated Lp(a), and the degree of lowering level achieved with these agents is generally insufficient to normalize very high concentrations.

Thus, PCSK9 inhibitors currently represent a pragmatic therapeutic option for optimizing LDL-C levels while providing a modest ancillary reduction in Lp(a), pending the availability of dedicated Lp(a)-targeted therapies capable of achieving larger and sustained reductions (6, 104, 112).

### 7.5 Other available pharmacological therapies affecting Lp(a)

Several conventional therapies modestly reduce Lp(a) concentrations, but their limited efficacy and lack of dedicated outcome evidence preclude their consideration as specific Lp(a)-targeted treatments (5, 80).

Inclisiran, a small interfering RNA targeting hepatic PCSK9 synthesis, is approved for LDL-C lowering and has shown a modest ancillary effect on Lp(a), typically in the range of approximately 19–22% across clinical studies. Dose-ranging and phase-based programs suggest small-to-moderate reductions that are directionally similar to those observed with PCSK9 monoclonal antibodies, although generally insufficient to normalize markedly elevated Lp(a) concentrations (112–115).

Bempedoic acid, an ATP-citrate lyase inhibitor, does not meaningfully reduce Lp(a). Available trial-level and meta-analytic evidence indicates an overall neutral effect, suggesting that its clinical value in patients with elevated Lp(a) lies in LDL-C/apoB lowering and reduction of inflammatory burden, as reflected by hsCRP, rather than in Lp(a) modification (116, 117).

Aspirin has been associated with an approximately 20% reduction in Lp(a) in some reports, although this signal requires confirmation and does not constitute an indication for aspirin use per se (5). Data on its efficacy for primary prevention in reducing CAD risk remain controversial. Although current meta-analytic evidence has not demonstrated a cardiovascular benefit and suggests an increased bleeding risk, aspirin may still provide a potential advantage in carefully selected high-risk individuals (118–120).

Other agents may also exert modest effects on circulating Lp(a) concentrations, although none can currently be considered an evidence-based Lp(a)-lowering strategy (5). Niacin has consistently been shown to reduce Lp(a), with placebo-controlled trial meta-analyses reporting mean reductions of approximately 20–25%, despite substantial interindividual variability (121). However, large outcome trials conducted in statin-treated or otherwise high-risk populations failed to demonstrate incremental cardiovascular benefit and documented excess adverse events, thereby limiting its role in contemporary practice (122). Accordingly, niacin is best regarded as a legacy therapy that lowers Lp(a) biochemically but lacks outcome validation and has an unfavorable risk–benefit profile compared with emerging targeted approaches (123).

Estrogen/progestin therapy may reduce Lp(a) by approximately 15%, with reductions in the range of 17–23% reported in older trials, but it cannot be considered a therapeutic option for elevated Lp(a) because of its broader atherothrombotic and clinical risk trade-offs (124, 125). Thyroid hormone analogs such as eprotirome have shown Lp(a) reductions of up to approximately 43%, but they are not approved and lack cardiovascular outcome data (126).

Lomitapide, used primarily for homozygous familial hypercholesterolemia, appears to have a modest and variable effect on Lp(a), with some reports describing reductions of approximately 15–20%, whereas other real-world analyses suggest little or no consistent change. It should therefore not be considered a reliable Lp(a)-directed therapy (127, 128). Mipomersen, a legacy agent not approved by the EMA, has shown an approximately 26% reduction in Lp(a) in clinical trials, but safety and tolerability concerns have prevented broader clinical use (129). By contrast, fibrates and omega-3 fatty acids do not appear to exert clinically relevant effects on Lp(a) concentrations (117).

Cholesteryl ester transfer protein (CETP) inhibitors, which are also not approved, may lower Lp(a) by approximately 25–40%; although mechanistic studies demonstrated substantial reductions, including approximately 34–40% with anacetrapib, clinical benefit has not been established for this class (130, 131). Interestingly, obicetrapib has shown consistent reductions in both LDL-C and Lp(a). In a recent pooled analysis of 2,356 patients with heterozygous familial hypercholesterolemia or established atherosclerotic cardiovascular disease treated with obicetrapib 10 mg once daily for 12 weeks, placebo-adjusted reductions of 37.0% in LDL-C, 21.3% in apolipoprotein B, and 37.3% in Lp(a) were observed, corresponding to an absolute Lp(a) reduction of 14.9 nmol/L. The greatest relative reduction in Lp(a) was achieved in patients with baseline concentrations between 50 and <150 nmol/L (−43.3%; −36.3 nmol/L). Although patients with baseline Lp(a) ≥ 150 nmol/L experienced a smaller proportional reduction, the absolute decrease remained comparable (−32.3 vs. −36.3 nmol/L), indicating a clinically relevant effect across a broad spectrum of baseline Lp(a) concentrations (132). While the magnitude of Lp(a) lowering is substantially lower than that achieved with RNA-targeted therapies, obicetrapib may represent an attractive option for patients with mild-to-moderately elevated Lp(a), particularly those who are unlikely to meet eligibility criteria for dedicated Lp(a)-lowering agents. Although its lipid-lowering profile is promising, its impact on cardiovascular outcomes remains to be established. This question is currently being addressed by the ongoing phase 3 PREVAIL trial (NCT05202509), a randomized cardiovascular outcomes study which has completed enrollment of 9,541 patients with established ASCVD and is expected to complete follow-up in November 2026 (133). Moreover, in the Multi-Ethnic Study of Atherosclerosis (MESA), regular aspirin use was evaluated in relation to Lp(a) and CAVS, supporting the hypothesis that antithrombotic and anti-inflammatory modulation may be particularly relevant in individuals with elevated Lp(a). However, this study has important limitations: its observational design, aspirin exposure was not randomized and may have been misclassified over time, and CAVS assessment relied on imaging-based endpoints rather than hard clinical outcomes such as valve replacement; accordingly, these observations should be regarded as hypothesis-generating rather than practice-changing. Nevertheless, the data provide additional biological plausibility to the concept that targeting pathways downstream of Lp(a), including thrombotic and inflammatory signaling, may influence the development or progression of valvular calcification. Dedicated outcome trials of Lp(a)-lowering therapies will be necessary to determine whether modifying the Lp(a) axis can produce disease-modifying effects on CAVS progression (134). Taken together, these therapies highlight the historical difficulty of modifying Lp(a) with conventional or repurposed pharmacologic strategies and further emphasize the need for dedicated approaches specifically designed to target the Lp(a) pathway.

### 7.6 Lipoprotein apheresis

Lipoprotein apheresis remains the most potent currently available intervention for lowering Lp(a) concentrations, producing acute reductions of approximately 60–75% per treatment session, with partial rebound between procedures depending on treatment intervals and baseline Lp(a) production rates (135, 136). Because apheresis also lowers LDL-C and other apoB-containing lipoproteins, it is typically reserved for highly selected patients with severe hyperlipoproteinemia, often including markedly elevated Lp(a) and progressive or recurrent ASCVD despite maximally tolerated medical therapy (136, 137). Prospective observational multicenter studies have demonstrated the feasibility of long-term apheresis programs in this population and suggested reductions in cardiovascular event rates during follow-up compared with the pre-apheresis period. Additional studies have further characterized the clinical phenotype of patients most likely to benefit from this intervention, typically those with progressive cardiovascular disease, markedly elevated Lp(a), and persistent events despite optimized medical therapy (137, 138). Moreover, as previously discussed, apheresis may attenuate CAVS progression in high-risk patients (52). Nevertheless, the invasive nature of the procedure, the need for repeated treatments, high costs, and limited availability confine lipoprotein apheresis to specialized centers and carefully selected clinical scenarios (135, 139, 140). Apheresis is often considered a niche or bridging strategy for extreme-risk patients while the field awaits outcome data from dedicated pharmacologic Lp(a)-lowering therapies capable of achieving substantial and sustained reductions without procedural burden (6, 104).

## 8 Emerging RNA-based therapies: targeting Lp(a) at its source

**Figure 2:**
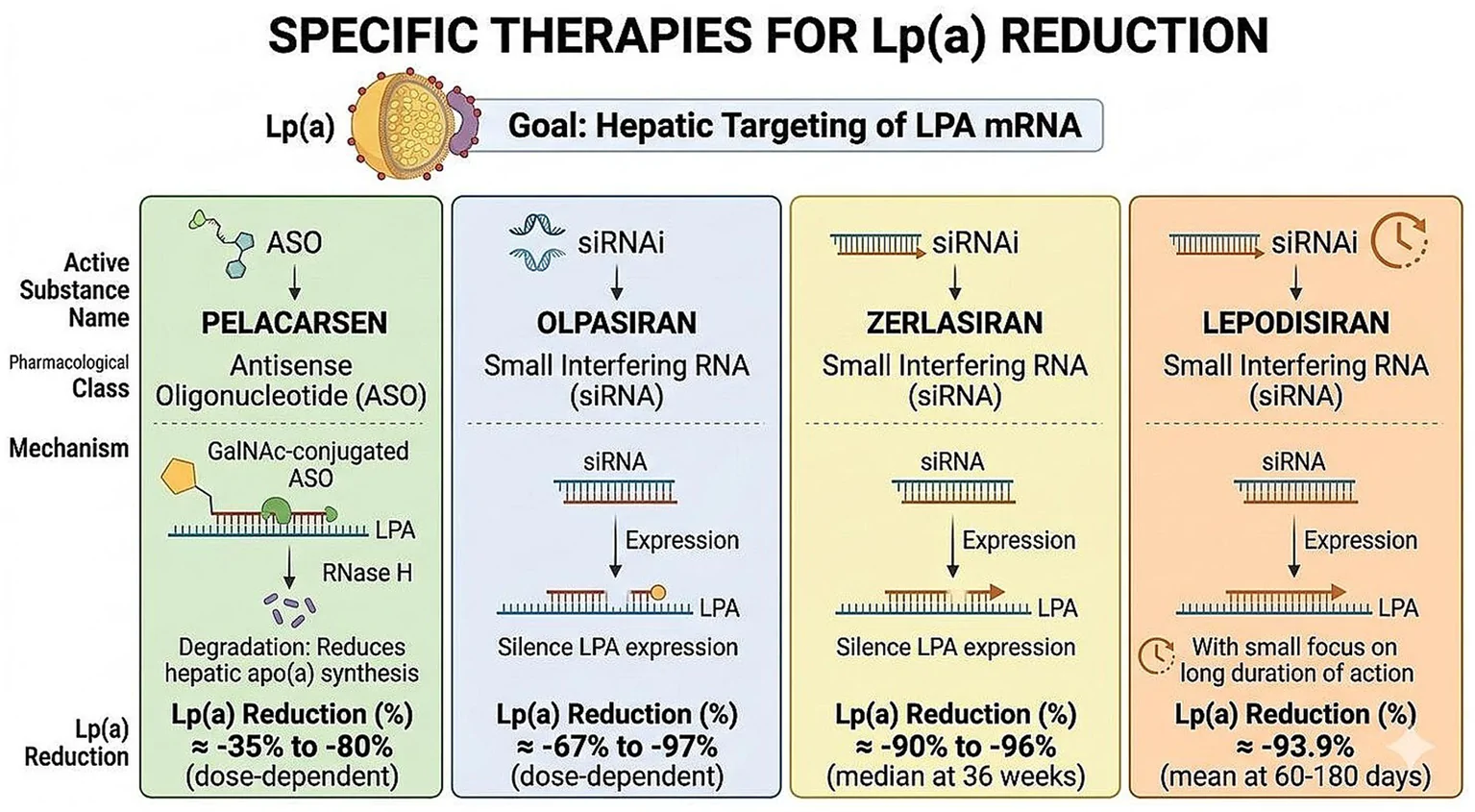
RNA-targeted therapies for Lp(a) lowering: principal agents and mechanisms of action. This figure summarizes the main RNA-targeted therapeutic approaches currently under clinical development for lipoprotein(a) [Lp(a)] reduction, including pelacarsen, olpasiran, zelasiran, and lepodisiran. These agents act by selectively suppressing hepatic apolipoprotein(a) synthesis through distinct nucleic acid–based strategies, including antisense oligonucleotide and small interfering RNA platforms. By targeting *LPA* gene expression upstream, these therapies achieve profound and sustained reductions in circulating Lp(a) concentrations and represent the most advanced pharmacological approaches currently being investigated to address Lp(a)-mediated residual cardiovascular risk.

The development of RNA-based therapeutics represents a major paradigm shift in the management of elevated Lp(a). These strategies include two complementary therapeutic platforms: antisense oligonucleotides (ASOs) and small interfering RNAs (siRNAs). Although both approaches selectively reduce hepatic apolipoprotein(a) production by targeting *LPA* messenger RNA, they differ in their molecular mechanisms of action. ASOs promote RNase H-mediated degradation of *LPA* mRNA, whereas siRNAs induce sequence-specific mRNA cleavage through the RNA-induced silencing complex (RISC). Both platforms have demonstrated profound reductions in circulating Lp(a) concentrations and are currently being evaluated in advanced clinical development. These agents directly suppress hepatic apo(a) production by targeting *LPA* expression, thereby addressing the upstream determinant of circulating Lp(a) rather than attempting to mitigate only the downstream consequences of its atherogenic, inflammatory, thrombotic, and pro-calcific effects (80, 105). Among emerging Lp(a)-lowering strategies, RNA-targeted agents, including pelacarsen, olpasiran, zerlasiran, and lepodisiran, have demonstrated the greatest specificity and magnitude of effect. By acting at the level of hepatic apo(a) synthesis, these therapies achieve marked and sustained reductions in circulating Lp(a) concentrations and represent the most advanced pharmacological approaches currently under clinical investigation (Figure 2).

### 8.1 Antisense oligonucleotides (ASO)

Pelacarsen is a GalNAc-conjugated antisense oligonucleotide that binds *LPA* mRNA and promotes RNase H–mediated degradation, thereby reducing apo(a) synthesis in hepatocytes (3, 11). Early dose-ranging ASO studies targeting apo(a) already demonstrated substantial mean reductions in Lp(a), in the range of approximately 67–72%, providing strong proof-of-concept for transcript-level suppression (141). In a phase 2 trial enrolling patients with established cardiovascular disease and elevated Lp(a), pelacarsen produced clear dose- and schedule-dependent reductions, with least-squares mean percent changes from baseline to 6 months ranging from approximately −35% with 20 mg every 4 weeks to approximately −80% with 20 mg weekly, approaching 90% in some individuals (104). Beyond lowering Lp(a) concentrations, mechanistic analyses suggest that pelacarsen may also reduce OxPLs and attenuate inflammatory and thrombotic signaling linked to Lp(a), potentially amplifying its biological effect beyond biomarker reduction alone (80). The ongoing phase 3 Lp(a) HORIZON trial is designed to determine whether pelacarsen-mediated Lp(a) lowering translates into a reduction in MACEs in patients with established ASCVD and elevated Lp(a) (142).

### 8.2 Small interfering RNA (siRNA)

SiRNA therapies including olpasiran, zerlasiran, and longer-duration siRNA lepodisiran, reduce Lp(a) concentrations through selective post-transcriptional silencing of hepatic *LPA* expression. After cellular uptake into hepatocytes, the double-stranded siRNA is incorporated into the RNA-induced silencing complex (RISC), where the passenger strand is removed and the guide strand directs sequence-specific recognition of *LPA* messenger RNA; the Argonaute-2 catalytic component of RISC subsequently cleaves the target mRNA, leading to its degradation and suppression of apolipoprotein(a) synthesis. Because the activated RISC complex can repeatedly target and degrade multiple mRNA transcripts, siRNA-based therapies may achieve profound and sustained reductions in circulating Lp(a) levels with infrequent dosing regimens (18, 143, 144).

This strategy is particularly compelling because Lp(a) concentrations are largely genetically determined and only minimally influenced by currently available lipid-lowering therapies, and siRNA agents have shown to determine profound and sustained reductions in Lp(a), often exceeding 90%, with infrequent dosing schedules that make them particularly attractive for long-term cardiovascular prevention (80).

Olpasiran (AMG 890) was evaluated in the phase 2 OCEAN(a)-DOSE trial, which tested multiple dosing regimens in patients with ASCVD and baseline Lp(a) concentrations >150 nmol/L. Placebo-adjusted reductions at week 36 were marked and dose-dependent, reaching approximately −67% with 10 mg every 12 weeks, −94% with 75 mg every 12 weeks, and approximately −97% with 225 mg every 12 or 24 weeks (18). Importantly, off-treatment follow-up demonstrated sustained Lp(a) suppression after discontinuation, suggesting prolonged pharmacodynamic effects that may be advantageous in long-term prevention strategies (145).

Zerlasiran (SLN360) initially showed dose-dependent Lp(a) lowering in first-in-human single ascending-dose studies, supporting both deep pharmacodynamic suppression and acceptable early tolerability (146). More recently, it demonstrated robust time-averaged Lp(a) lowering in a randomized clinical trial in stable ASCVD. Least-squares mean time-averaged reductions through 36 weeks were approximately 81–86% across dosing regimens, with median week-36 reductions typically ranging from approximately 90 to 96%. Injection-site reactions were the main treatment-related adverse event and were generally mild (147).

Lepodisiran (LY3819469), evaluated in the ALPACA program, further extends this concept by aiming for an even longer duration of action. In a randomized trial, placebo-adjusted time-averaged changes in Lp(a) from day 60 to day 180 reached −93.9% in pooled 400-mg groups, with durable suppression extending up to 12 months depending on the dosing schedule. Its safety profile was dominated by generally mild injection-site reactions (148).

### 8.3 Comparative pharmacology: ASO vs. siRNA

Antisense oligonucleotides and siRNA therapies both reduce apo(a) production by targeting *LPA* mRNA, but they differ in intracellular mechanism and, in practical terms, in dosing cadence and duration of effect (105, 147, 148). Pelacarsen, as an ASO, binds *LPA* mRNA and promotes RNase H–mediated degradation. In phase 2 studies, meaningful lowering required relatively frequent administration, with the greatest effects observed under weekly dosing regimens, and a clear dose-dependent relationship was observed (3, 142). By contrast, siRNA agents such as olpasiran, zerlasiran, and lepodisiran are incorporated into the RISC complex and silence *LPA* through the RNA interference pathway. Across trials, these compounds have consistently produced deep reductions in Lp(a), often ≥90%, with infrequent administration schedules, including every 12 or 24 weeks or, in some settings, even longer intervals. Persistent off-treatment suppression has been a particularly notable feature of this platform (18, 145). Across both therapeutic platforms, the most common short-term adverse events have been injection-site reactions, usually mild, and occasional flu-like symptoms. Importantly, early studies suggest a high degree of target specificity, without clinically relevant adverse spillover effects on other lipid fractions (80, 105, 146, 147).

Collectively, early-phase data indicate that RNA-based therapies can achieve very large, selective, and sustained reductions in Lp(a), often in the range of 80–90% or greater, with limited impact on other lipid fractions and acceptable short-term tolerability profiles (142, 146–148). These findings strongly support the mechanistic rationale for RNA-targeting approaches and position them as the most promising class of Lp(a)-directed therapies currently under development. The two major RNA-based strategies for selective Lp(a) lowering, antisense oligonucleotides and small interfering RNA therapies, are compared in Table 5.

**Table 5:** RNA-based therapies targeting lipoprotein(a): mechanistic and clinical characteristics.

Feature	Antisense oligonucleotides (ASO)	siRNA therapies
Molecular target	*LPA* mRNA	*LPA* mRNA
Mechanism of action	RNase H–mediated degradation of *LPA* mRNA	RNA interference via RISC complex
Site of action	Hepatocyte cytoplasm	Hepatocyte cytoplasm
Representative agents	Pelacarsen	Olpasiran, SLN360, Zerlasiran
Dosing frequency	Monthly subcutaneous administration	Every 3–6 months subcutaneous administration
Magnitude of Lp(a) reduction	~70–90%	~80–98%
Onset of effect	Gradual	Rapid and sustained
Duration of effect	Requires repeated dosing	Prolonged suppression after dosing
Potential advantage	Extensive clinical experience	Long dosing interval and greater durability
Main open issue	Cardiovascular outcome confirmation	Long-term safety and outcome validation

Comparison of the main RNA-based therapeutic platforms for selective Lp(a) lowering, highlighting their mechanisms of action, pharmacological characteristics, and clinical features. ASO, antisense oligonucleotide; GalNAc, N-acetylgalactosamine; Lp(a), lipoprotein(a); mRNA, messenger RNA; RNase H, ribonuclease H; RISC, RNA-induced silencing complex; siRNA, small interfering RNA.

### 8.4 From biomarker lowering to outcomes: phase 3 programs

The next critical step for the management of LP(a) mediated risk is outcome validation of therapeutic agents in development. The central unresolved question is whether large reductions in Lp(a) will translate into fewer cardiovascular events (149). Pelacarsen is currently being evaluated in the phase 3 Lp(a)HORIZON trial for reduction of MACEs (142, 150). Similarly, the phase 3 OCEAN(a)-Outcomes program is designed to assess whether olpasiran reduces coronary heart disease death, myocardial infarction, and urgent revascularization in patients with established ASCVD and elevated Lp(a), although composite endpoint definitions may vary across protocol versions (151).

ACCLAIM-Lp(a) is an ongoing phase 3, randomized, double-blind, placebo-controlled, event-driven cardiovascular outcomes trial evaluating whether lepodisiran reduces MACEs in adults with Lp(a) ≥ 175 nmol/L (approximately ≥70 mg/dL) and either established ASCVD or high risk for a first cardiovascular event. The study plans to enroll approximately 12,500 randomized participants and will evaluate a dosing regimen consisting of an initial administration phase at baseline, 6 months, and 12 months, followed by annual maintenance dosing thereafter, reflecting the prolonged durability of Lp(a) lowering observed in phase 2 studies. The primary endpoint is time to first occurrence of a four-component MACE (MACE-4), including cardiovascular death, non-fatal myocardial infarction, non-fatal stroke, or urgent coronary revascularization (152).

The field is rapidly expanding, reflecting the broader transition from viewing Lp(a) as a non-modifiable risk marker to recognizing it as a treatable target. The results of these trials will be essential to define the clinical positioning of Lp(a)-lowering therapies, identify the patients most likely to benefit, and determine how these agents should be integrated into future prevention strategies.

## 9 Emerging non-RNA therapies: beyond nucleic acid-based approaches

While most therapeutic advances in the Lp(a) field currently rely on antisense oligonucleotides and siRNA technologies, emerging strategies are exploring non–RNA-based approaches. Rather than suppressing hepatic *LPA* expression, these strategies aim to interfere with Lp(a) particle formation through alternative pharmacological mechanisms. Expanding the therapeutic landscape beyond nucleic acid-based approaches may prove important in the long term, particularly with respect to treatment acceptability, route of administration, and broader implementation in chronic prevention settings (5, 62, 80).

### 9.1 Oral small-molecule inhibition of Lp(a) assembly: muvalaplin

Muvalaplin is the first orally active small-molecule inhibitor specifically developed to reduce Lp(a) by preventing particle assembly rather than silencing *LPA* expression. Mechanistically, it disrupts the interaction between apo(a) and apoB100 required for Lp(a) formation, thereby representing a pharmacologically distinct strategy from ASO and siRNA therapies. This approach is particularly attractive because it introduces the possibility of a non-injectable treatment option for elevated Lp(a).

In a first-in-human randomized clinical trial, short-term administration of muvalaplin for 14 days resulted in rapid and dose-dependent Lp(a) lowering, with placebo-adjusted reductions of approximately 63–65%, while no clinically meaningful effects on plasminogen activity were observed. Overall tolerability was favorable in this early-phase setting, providing proof-of-concept for oral pharmacologic inhibition of Lp(a) formation (153). More recently, a randomized phase 2 trial evaluated muvalaplin in high-risk individuals with elevated Lp(a), including patients with established ASCVD, diabetes, or familial hypercholesterolemia. After 12 weeks of treatment, muvalaplin significantly reduced Lp(a) levels compared with placebo. The extent of reduction varied depending on the dose and the assay used, with the greatest decreases observed when measured using intact-particle assays, and good tolerability (154). These data further support the biological efficacy of muvalaplin and reinforce the concept that non-RNA pharmacological inhibition of Lp(a) may become a clinically relevant complement to injectable gene-silencing strategies. However, cardiovascular outcome data are not yet available, and its long-term safety, durability, and eventual clinical positioning remain to be established.

From a broader perspective, oral small-molecule inhibition may become particularly attractive within future prevention pathways, especially for patients requiring long-term treatment acceptance beyond conventional LDL-C lowering. In this context, muvalaplin may represent an important expansion of the Lp(a)-lowering armamentarium, although other studies are needed to determine whether its effects on LP(a) levels translate into cardiovascular benefit.

Table 6 summarizes the main clinical evidence on muvalaplin as the first oral non-RNA therapy designed to lower Lp(a) by inhibiting lipoprotein(a) particle assembly.

**Table 6:** Muvalaplin: key clinical trial evidence as an emerging non–RNA-based Lp(a)-lowering therapy.

Modality	Key trial(s)	Population (core)	Dosing tested	Lp(a) lowering (key quantitative signal)	Durability	Main early safety signals
Oral small molecule inhibitor of Lp(a) formation	Phase 1 randomized clinical trial, 2023; Phase 2 randomized clinical trial, 2025	Phase 1: healthy participants/individuals with elevated Lp(a); Phase 2: adults with Lp(a) ≥ 175 nmol/L and established ASCVD, diabetes, or familial hypercholesterolaemia	Phase 1: daily oral administration for 14 days; Phase 2: 10 mg, 60 mg, or 240 mg once daily for 12 weeks	Phase 1: placebo-adjusted Lp(a) reduction up to ~63–65%; Phase 2: placebo-adjusted reductions by intact Lp(a) assay of 47.6, 81.7, and 85.8% with 10, 60, and 240 mg/day, respectively; corresponding reductions by apo(a)-based assay were 40.4, 70.0, and 68.9%	On-treatment efficacy demonstrated; requires continued daily oral dosing; long-term persistence after discontinuation remains to be defined	Generally well tolerated in early trials; no clinically meaningful plasminogen signal reported in phase 1; no major short-term safety concerns identified

Summary of the main clinical trials evaluating muvalaplin, including study design, patient population, dosing regimens, efficacy, durability of Lp(a) lowering, and early safety findings. Percent reductions in Lp(a) with muvalaplin may differ according to the assay used, particularly when comparing intact-particle assays with apo(a)-based assays. Apo(a), apolipoprotein(a); ApoB100, apolipoprotein B100; ASCVD, atherosclerotic cardiovascular disease; LPA, lipoprotein(a) gene; Lp(a), lipoprotein(a).

## 10 Future perspectives

Despite the remarkable progress in understanding Lp(a) biology and the rapid development of targeted therapies, several clinically relevant questions remain unresolved. These knowledge gaps will likely influence future patient selection, therapeutic strategies, and the integration of Lp(a)-lowering therapies into clinical practice and guideline recommendations. Key unresolved issues regarding the clinical management and therapeutic targeting of lipoprotein(a) are summarized in Table 7.

**Table 7:** Unresolved clinical and research questions in Lp(a) management.

Domain	Open question	Clinical relevance
Risk stratification	What is the optimal Lp(a) threshold for treatment initiation?	Patient selection
Primary prevention	Should high Lp(a) be treated in the absence of established ASCVD?	Cost–benefit balance
Secondary prevention	Should Lp(a)-lowering therapies be used as add-on or phenotype-driven strategies?	Therapeutic sequencing
Valvular disease	Can Lp(a) lowering slow the progression of calcific aortic stenosis?	Disease-modifying potential
Timing of intervention	Is earlier treatment more effective than late intervention?	Lifetime risk reduction
Combination therapy	Can Lp(a) lowering synergize with intensive LDL-C reduction?	Residual risk minimization
Guidelines integration	How should Lp(a) be incorporated into future risk algorithms and treatment pathways?	Future guideline implementation

Summary of the major unresolved issues regarding risk stratification, patient selection, therapeutic strategies, and future clinical implementation of Lp(a)-lowering therapies. ASCVD, atherosclerotic cardiovascular disease; LDL-C, low-density lipoprotein cholesterol; Lp(a), lipoprotein(a).

## 11 Conclusion

Lipoprotein(a) is increasingly recognized as a genetically determined factor potentially associated with residual cardiovascular risk in both ASCVD and CAVS. Its pathophysiological effects extend well beyond cholesterol transport and include pro-inflammatory, pro-thrombotic, and pro-calcific mechanisms, supporting the possibility that Lp(a) may be more than a biomarker of risk, potentially contributing to disease through biological activity.

A major therapeutic transition is now underway. For decades, elevated Lp(a) represented a clinically important but largely non-modifiable risk factor, insufficiently addressed by conventional lipid-lowering therapies. The development of RNA-based agents capable of producing profound and sustained reductions in circulating Lp(a), together with emerging non-RNA strategies such as oral small-molecule inhibitors, may change this landscape. These advances raise the realistic prospect that Lp(a) may soon become a treatable target in routine cardiovascular prevention.

At present, measurement of Lp(a) at least once during adult life represents a practical and increasingly supported strategy to improve cardiovascular risk stratification, particularly in individuals with premature ASCVD, recurrent events despite optimal LDL-C control, familial hypercholesterolemia, or early calcific aortic valve disease. Identifying elevated Lp(a) has immediate clinical value because it refines risk estimation, strengthens the rationale for aggressive management of all modifiable risk factors, and may help identify patients most likely to benefit from future Lp(a)-directed therapies.

An important remaining question is whether Lp(a) is causally involved in cardiovascular disease and whether substantial Lp(a) lowering may translate into improved clinical outcomes. The results of ongoing phase 3 trials will therefore be pivotal. They will determine the magnitude of benefit achievable with Lp(a)-directed treatment, clarify the most appropriate thresholds for intervention, and define how these therapies should be integrated into future prevention algorithms. If these trials are positive, they are likely to reshape the management of inherited residual cardiovascular risk and open a new era in precision preventive cardiology.
